# Association between physicians’ characteristics and their knowledge, attitudes, and practices regarding advance care planning: a cross-sectional study

**DOI:** 10.1186/s12904-023-01253-x

**Published:** 2023-09-11

**Authors:** Ayaka Sakamoto, Ryota Inokuchi, Masao Iwagami, Kyoko Hanari, Nanako Tamiya

**Affiliations:** 1 Health Services Research and Development Center, University of Tsukuba, 1-1-1 Tenno-Dai, Tsukuba, Ibaraki 305-8577 Japan; 2 Department of Health Services Research, Graduate School of Comprehensive Human Sciences, University of Tsukuba, 1-1-1 Tenno-Dai, Tsukuba, Ibaraki 305-8577 Japan; 3 Department of Health Services Research, Institute of Medicine, University of Tsukuba, 1-1-1 Tenno-Dai, Tsukuba, Ibaraki 305-8577 Japan

**Keywords:** Advance care planning, Terminal care, Physicians, Japan, Cross-sectional study

## Abstract

**Background:**

Despite physicians’ vital role in advance care planning, a limited number of physicians practice it. This study assessed factors associated with physicians’ knowledge, attitudes, and practices regarding advance care planning.

**Methods:**

This cross-sectional study used data from an anonymous survey conducted by the Japanese Ministry of Health, Labour and Welfare.

Questionnaires were mailed to 4500 physicians in November and December 2022. Data from 1260 respondents were analyzed.

**Results:**

Of the respondents, 46.4%, 77.0%, and 82.0% reported good knowledge of advance care planning, agreed with promoting it, and with its provision by medical/care staff, respectively. Male physicians were significantly less likely to support advance care planning (odds ratio: 0.54, 95% confidence interval: 0.35–0.84) or agree to its provision by medical/care staff (odds ratio: 0.47, 95% confidence interval: 0.29–0.78) but significantly more likely to practice it (odds ratio: 1.58, 95% confidence interval: 1.05–2.36). Physicians specialized in surgery or internal/general/palliative medicine were more knowledgeable about advance care planning and more likely to practice it. Physicians working in clinics were significantly less knowledgeable (odds ratio: 0.33, 95% confidence interval: 0.25–0.44) about advance care planning and less likely to support it (odds ratio: 0.37, 95% confidence interval: 0.27–0.50), agree with its provision by medical/care staff (odds ratio: 0.54, 95% confidence interval: 0.39–0.75), or to practice it (odds ratio: 0.16, 95% confidence interval: 0.12–0.22).

**Conclusions:**

Physicians working in clinics had less knowledge of advance care planning, less supportive attitudes, and less likely to practice it. Knowledge, attitudes and practice also varied by gender and specialty. Interventions should target physicians working in clinics.

**Supplementary Information:**

The online version contains supplementary material available at 10.1186/s12904-023-01253-x.

## Background

Advance care planning is a process for setting life goals and planning medical care based on one’s goals, values, and preferences.^1^ Physicians play an important role in this process by translating patients’ broad viewpoints into specific discussions regarding their medical care preferences [1]. However, there is a limited number of physicians who practice advance care planning [2, 3].

Previous studies reported that the physician factors associated with advance care planning include older age, being a woman, having experience of advance care planning, and caring for dying patients [2, 4–7]. The place of medical training was not associated with advance care planning [4, 8]. Meanwhile, the place of practice and years of practice and education have shown contradictory results among studies [2, 4, 6–8]. These studies also limited participants with specific specialties [4, 5, 8], had mixed participants of physicians and nurses [6], and mainly assessed physicians experience [2, 7]; thus the association between physician characteristics and advance care planning remains unclear.

Information on physician characteristics associated with the knowledge, attitudes, and practice regarding advance care planning would be useful for promoting it among physicians. Therefore, this study aimed to assess the association between physicians’ characteristics and their knowledge, attitudes, and practice regarding advance care planning, using the results of a nationwide survey including physicians from various backgrounds.

## Methods

### Participants and procedures

We conducted a cross-sectional study using data from a nationwide anonymous survey of physicians’ attitudes toward end-of-life medical care [9]. The survey was conducted by the Japanese Ministry of Health, Labour and Welfare approximately every five years since 1992. The data used in this study were obtained in November and December 2022. Questionnaires were mailed to randomly selected hospitals and clinics throughout Japan. Hospital managers were requested to select at least one physician involved in end-of-life medical care to complete the questionnaire. The survey was distributed to 4500 physicians: two physicians per hospital from 1500 hospitals, and one physician per clinic from 1500 clinics. The participants responded using a return envelope or web survey system.

Anonymized data were obtained from the Ministry of Health, Labour and Welfare. The requirement for informed consent was waived because providing a response was considered consent, and the study protocol was approved by the Ethics Committee, Institute of Medicine, University of Tsukuba (approval number: 1791, date of approval: September 2, 2022).

### Measures

As outcomes, we used one question regarding the respondents’ knowledge, two questions regarding attitudes, and one question regarding the practice of advance care planning. One of the attitude questions asked whether advance care planning should be promoted and the other asked whether it should be provided by medical/care staff. Practice was assessed with a question regarding the extent of discussions with patients regarding their end-of-life medical care. Details of the questionnaires and answers are described in Appendix 1.

The independent variables included respondent gender, years of practice, specialties, and workplace. We divided the 20 specialties into five categories namely internal medicine, general medicine, palliative care, surgery, and others. The variable years of practice was divided into three categories (≤ 15 years, 16–30 years, and ≥ 31 years), based on a previous study [2].

### Statistical analyses

We first conducted a descriptive analysis of all variables, followed by bivariate analyses using χ^2^ tests or Fisher’s exact test to evaluate the association between physician characteristics and their advance care planning knowledge, attitudes, and practices. Multivariable logistic regression analyses were conducted for each of the outcomes using the four categorical independent variables of gender, years of practice, specialties, and workplace. Respondents with missing data for key variables were excluded.

Furthermore, we conducted two sensitivity analyses: excluding respondents not involved in caring for patients in the end-of-life stage; and excluding respondents not involved in caring for patients in the end-of-life stage and including respondents who specialized in internal or general medicine.

Statistical analyses were conducted using Stata 15 (StataCorp, College Station, TX, USA). Two-sided *p* values < 0.05 were regarded as statistically significant.

## Results

**Fig. 1:**
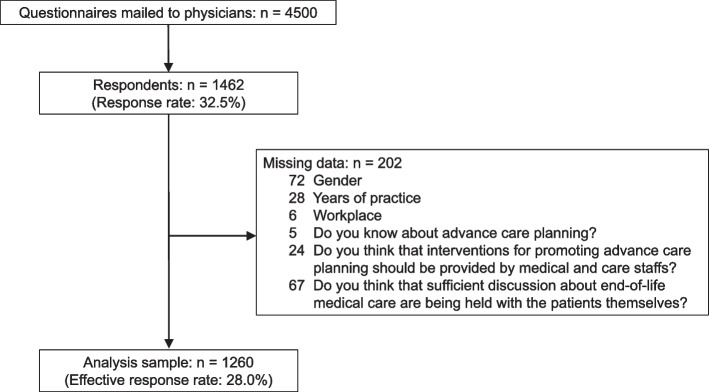
Flow chart of data collection

A total of 1462 completed questionnaires were returned (response rate: 32.5%). After excluding those with missing data, 1260 respondents were included in the analysis (effective response rate: 28.0%). Details of data collection are shown in Fig. 1.

### Characteristics of the respondents

Respondent characteristics are shown in Table 1. Of the respondents, 84.3% were men (compared with 77.2% of physicians in Japan in 2020) [10], and 50.7% had practiced for over 31 years. Of the respondents, 45.8%, 5.6%, 10.3%, and 17.1% selected internal medicine, general medicine, palliative care, and surgery as their specialties, respectively; 57.2% were working at hospitals, and 37.9% were working in clinics.

**Table 1:** Characteristics of the respondents (*N* = 1260)

	n	(%)
Sex		
Male	1062	(84.3)
Years of practice		
1–15	153	(12.1)
16–30	468	(37.1)
≥ 31	639	(50.7)
Specialty (multiple answers permitted)		
Internal medicine	577	(45.8)
General medicine	71	(5.6)
Palliative care	130	(10.3)
Surgery	216	(17.1)
Others	572	(45.4)
Workplace		
Hospital	721	(57.2)
Clinic	478	(37.9)
Nursing Home	17	(1.3)
Others	44	(3.5)
Good knowledge of ACP		
Yes	585	(46.4)
No	675	(53.6)
Believe that ACP should be promoted		
Agree	970	(77.0)
Disagree	17	(1.3)
Not sure	273	(21.7)
Believe that medical and care staff should provide ACP		
Necessary	1,035	(82.1)
Not necessary	43	(3.4)
Not sure	182	(14.4)
Extent of discussions about end-of-life medical care		
Sufficiently	229	(18.2)
To some extent	571	(45.3)
Infrequent	273	(21.7)
Not involved in providing end-of-life care	187	(14.8)

*ACP* Advance care planning

Only 46.4% of the respondents stated that they “know advance care planning well”; however, the majority agreed that advance care planning should be promoted and provided by medical/care staff (77% and 82.1%, respectively). Further, 63.5% reported that they discussed end-of-life care with their patients “sufficiently” (18.2%) or “to some extent” (45.3%).

### Knowledge of advance care planning

The associations between respondents’ characteristics and their knowledge of advance care planning are shown in Table 2. Physicians’ specialties and workplace were significantly associated with their knowledge of advance care planning. Physicians who specialized in internal medicine, general medicine, palliative care, and surgery were knowledgeable about advance care planning (odds ratio [OR]: 1.85, 95% confidence interval [CI]: 1.29–2.64; OR: 6.73, 95% CI: 3.29–13.78; and OR: 15.23, 95% CI: 7.42–31.26; and OR: 1.56, 95% CI: 1.04–2.35, respectively). Physicians working in clinics and nursing homes had significantly less knowledge (OR: 0.33, 95% CI: 0.25–0.44; and OR: 0.33, 95% CI: 0.11–0.97, respectively) than those working at other workplaces. Physicians’ gender and years of practice were not associated with their knowledge of advance care planning.

**Table 2:** Association between physicians’ characteristics and good knowledge of advance care planning

	Good knowledge of advance care planning			
	Bivariate analysis			Multivariable logistic regression analysis
	Yes (*N* = 585) n (%)	No (*N* = 675) n (%)	p	OR [95% CI]
Gender			0.897	
Male	493 (46.4)	569 (53.6)		1.05 [0.72–1.52]
Years of practice			0.118	
1–15	79 (51.6)	74 (48.4)		1.00 (ref)
16–30	227 (48.5)	241 (51.5)		1.26 [0.82–1.95]
≥ 31	279 (43.7)	360 (56.3)		1.22 [0.79–1.86]
Specialty				
Internal medicine	294 (50.9)	283 (49.1)	0.003	1.85 [1.29–2.64]
General medicine	61 (85.9)	10 (14.1)	< 0.001	6.73 [3.29–13.78]
Palliative care	121 (93.1)	9 (7.0)	< 0.001	15.23 [7.42–31.26]
Surgery	127 (58.8)	89 (41.2)	< 0.001	1.56 [1.04–2.35]
Others	192 (33.6)	380 (66.4)	< 0.001	0.77 [0.54–1.09]
Workplace				
Hospital	434 (60.2)	287 (39.8)	< 0.001	1.00 (ref)
Clinic	121 (25.3)	357 (74.7)	< 0.001	0.33 [0.25–0.44]
Nursing home	5 (29.4)	12 (70.6)	0.16	0.33 [0.11–0.97]
Others	25 (56.8)	19 (43.2)	0.16	1.34 [0.69–2.61]

*CI* Confidence interval, *OR* Odds ratio

### Promotion of advance care planning

The associations between respondents’ characteristics and their attitudes toward the promotion of advance care planning are shown in Table 3. Male physicians and those working in clinics were less likely to support the promotion of advance care planning (OR: 0.54, 95% CI: 0.35–0.84; and OR: 0.37, 95% CI: 0.27–0.50, respectively), whereas physicians specialized in internal medicine were more likely to support it (OR: 1.80, 95% CI: 1.21–2.69). Years of practice was not associated with physicians’ attitude to the promotion of advance care planning.

**Table 3:** Association between physicians’ characteristics and agreement that advance care planning should be promoted

	Agree that advance care planning be promoted			
	Bivariate analysis			Multivariable logistic regression analysis
	Yes (*n* = 970) n (%)	No/Not sure (*n* = 290) n (%)	p	OR [95%CI]
Gender			0.007	
Male	803 (75.6)	259 (24.4)		0.54 [0.35–0.84]
Years of practice			0.009	
1–15	132 (86.3)	21 (13.7)		1.00 (ref)
16–30	361 (77.1)	107 (22.9)		0.66 [0.39–1.12]
≥ 31	477 (74.7)	162 (25.4)		0.68 [0.40–1.14]
Specialty				
Internal medicine	467 (80.9)	110 (19.1)	0.002	1.80 [1.21–2.69]
General medicine	64 (90.1)	7 (9.9)	0.007	1.95 [0.86–4.39]
Palliative care	116 (89.2)	14 (10.8)	< 0.001	1.80 [0.97–3.33]
Surgery	177 (81.9)	39 (18.1)	0.057	1.42 [0.89–2.27]
Others	407 (71.2)	165 (28.8)	< 0.001	1.02 [0.68–1.54]
Workplace				
Hospital	613 (85.0)	108 (15.0)	< 0.001	1.00 (ref)
Clinic	305 (63.8)	173 (36.2)	< 0.001	0.37 [0.27–0.50]
Nursing home	16 (94.1)	1 (5.9)	0.14	2.97 [0.39–22.89]
Others	36 (81.8)	8 (18.2)	0.44	0.85 [0.38–1.91]

*CI* Confidence interval, *OR* Odds ratio

### *Provision of advance care planning by medical/care staff*

The associations between physicians’ characteristics and their attitudes toward provision of advance care planning by medical/care staff are shown in Table 4. Male physicians and physicians working in clinics were less likely to agree that advance care planning should be provided by medical/care staff (OR: 0.47, 95% CI: 0.29–0.78; and OR: 0.54, 95% CI: 0.39–0.75, respectively), whereas physicians specialized in palliative care were more likely to agree (OR: 2.43, 95% CI: 1.13–5.24). Years of practice was not associated with physicians’ attitude regarding provision of advance care planning by medical/care staff in the multivariable analysis.

**Table 4:** Association between physicians’ characteristics and agreement that medical/care staff should provide advance care planning

	Agree that advance care planning be provided by medical and care staff			
	Bivariate analysis			Multivariable logistic regression analysis
	Yes (*N* = 1035) n (%)	No/Not sure (*N* = 225) n (%)	p	OR [95%CI]
Gender			0.004	
Male	858 (80.8)	204 (19.2)		0.47 [0.29–0.78]
Years of practice			0.14	
1–15	134 (87.6)	19 (12.4)		1.00 (ref)
16–30	385 (82.3)	83 (17.7)		0.78 [0.45–1.36]
≥ 31	516 (80.8)	123 (19.2)		0.84 [0.48–1.45]
Specialty				
Internal medicine	484 (83.9)	93 (16.1)	0.14	1.20 [0.78–1.83]
General medicine	67 (94.4)	4 (5.6)	0.006	2.68 [0.95–7.55]
Palliative care	122 (93.9)	8 (6.2)	< 0.001	2.43 [1.13–5.24]
Surgery	191 (88.4)	25 (11.6)	0.008	1.52 [0.90–2.58]
Others	439 (76.8)	133 (23.3)	< 0.001	0.70 [0.46–1.09]
Workplace				
Hospital	628 (87.1)	93 (12.9)	< 0.001	1.00 (ref)
Clinic	350 (73.2)	128 (26.8)	< 0.001	0.54 [0.39–0.75]
Nursing home	16(94.1)	1 (5.9)	0.34	2.69 [0.35–20.78]
Others	41 (93.2)	3 (6.8)	0.052	2.38 [0.71–7.96]

*CI* Confidence interval, *OR* Odds ratio

### Advance care planning practices

The associations between physicians’ characteristics and advance care planning practices are shown in Table 5. Male physicians were more likely than female physicians to discuss end-of-life medical care with their patients (OR: 1.58, 95% CI: 1.05–2.36). Physicians’ years of practice and their advance care planning practices were associated in the bivariate analyses, but not in the multivariable analysis. Physician specialties were significantly associated with their advance care planning practices: those who specialized in internal medicine, general medicine, palliative care, and surgery were more likely to practice advance care planning (OR: 2.38, 95% CI: 1.60–3.55; OR: 3.38, 95% CI: 1.44–7.94; OR: 9.49, 95% CI: 3.67–24.53; and OR: 2.50, 95% CI: 1.55–4.05, respectively) than those with other specialties (OR: 0.56, 95% CI: 0.37–0.82). Physicians working in clinics were significantly less likely to practice advance care planning (OR: 0.16, 95% CI: 0.12–0.22) than those working in hospitals.

**Table 5:** Association between physicians’ characteristics and practice of advance care planning

	Practice advance care planning			
	Bivariate analysis			Multivariable logistic regression analysis
	Practice (*N* = 800) n (%)	No (*N* = 460) n (%)	p	OR [95%CI]
Gender			0.027	
Male	688 (64.8)	374 (35.2)		1.58 [1.05–2.36]
Years of practice			0.002	
1–15	113 (73.9)	40 (26.1)		1.00 (ref)
16–30	307 (65.6)	161 (34.4)		0.91 [0.56–1.49]
≥ 31	380 (59.5)	259 (40.5)		0.72 [0.44–1.16]
Specialty				
Internal medicine	419 (72.6)	158 (27.4)	< 0.001	2.38 [1.60–3.55]
General medicine	64 (90.1)	7 (9.9)	< 0.001	3.38 [1.44–7.94]
Palliative care	125 (96.2)	5 (3.8)	< 0.001	9.49 [3.67–24.53]
Surgery	180 (83.3)	36 (16.7)	< 0.001	2.50 [1.55–4.05]
Others	258 (45.1)	314 (54.9)	< 0.001	0.56 [0.37–0.82]
Workplace				
Hospital	591 82.0	130 (18.0)	< 0.001	1.00 (ref)
Clinic	167 34.9	311 (65.1)	< 0.001	0.16 [0.12–0.22]
Nursing home	14 82.4	3 (17.6)	0.10	1.16 [0.31–4.29]
Others	28 63.6	16 (36.4)	0.98	0.67 [0.33–1.36]

*CI* Confidence interval, *OR* Odds ratio, *Ref* Reference

### Sensitivity analyses

We conducted two sensitivity analyses: excluding respondents who were not involved in caring for patients in the end-of-life stage (*n* = 1073); and including respondents who specialized in internal or general medicine, excluding those not involved in caring for patients in the end-of-life stage (*n* = 567). The details are shown in Appendix 2. Similar to the main analysis, in the first sensitivity analysis, male physicians were significantly less likely to agree with the promotion of advance care planning or that it should be provided by medical/care staff; however, these differences were not significant in the second sensitivity analysis. As in the main analysis, years of practice was not associated with any outcome. The associations between specialties and outcomes were similar to the main analysis; however, some factors were no longer significant. In both sensitivity analyses, physicians working in clinics were significantly less likely than those working in hospitals to have a good knowledge of advance care planning, support it, agree it should be provided by medical/care staff, or practice it.

## Discussion

### Main findings

The study showed significant associations with gender, specialties, and workplace, but not with years of practice. Additionally, this study reveals the need to promote advance care planning which targets physicians working in clinics. To the best of our knowledge, this is the first national-level survey to assess the association between physicians’ characteristics and their knowledge, attitudes, and practice of advance care planning.

### Knowledge of advance care planning

Physicians who specialized in internal medicine, general medicine, palliative medicine, and surgery showed a higher knowledge of advance care planning. This may be because physicians in these specialties tend to be more involved in end-of-life care than those with other specialties.

Physicians working in clinics and nursing homes were less knowledgeable regarding advance care planning. This may be because physicians working in clinics do not often provide end-of-life care. However, even after excluding those not involved in end-of-life care in the sensitivity analyses, physicians working in clinics were still less knowledgeable. In cancer hospitals in Japan, it is necessary for physicians who care for cancer patients to participate in an education program about palliative care which mentions advance care planning as a component [11]. Therefore, physicians working in hospitals may have had more opportunities to receive education on advance care planning. The promotion of an education program for physicians working in clinics is desired.

The results regarding the association between physicians’ gender and advance care planning knowledge are inconsistent with a previous study that showed female medical staff were more knowledgeable about advance care planning [6]; however, this previous study did not distinguish between physicians and nurses and included few female physicians. When referring only to physicians, gender might not be associated with the knowledge of advance care planning.

### Promotion of advance care planning

Physicians’ gender, specialties, and workplace were associated with their agreement to promote advance care planning. It is unclear why, despite having the same degree of knowledge regarding advance care planning, male physicians are less likely than female physicians to agree to promote advance care planning. The sensitivity analysis excluding physicians not involved in end-of-life care found that the main results were robust. Therefore, further research is needed to examine the associations between physicians’ gender and attitude towards the promotion of advance care planning.

Physicians who specialized in internal medicine were more likely to support advance care planning than those with other specializations. This may be because they are more often involved in providing end-of-life care. Physicians working in clinics were significantly less likely to support promotion of advance care planning. This may be because they had less knowledge of advance care planning than those working elsewhere. Notably, most of the respondents who did not support the promotion of advance care planning answered they were “not sure” about it.

### *Provision of advance care planning by medical/care staff*

Physicians’ gender, specialty, and workplace were associated with their agreement that advance care planning should be provided by medical/care staff. A previous study has shown similar results that male physicians are less interested in advance care planning and less willing to practice it with their patients [5]; however, further research is needed to investigate the reason behind these gender differences.

Physicians who specialized in palliative care were most likely to agree with intervention by medical/care staff in advance care planning. This could be because their specialty focuses on end-of-life medical care. Furthermore, physicians working in clinics were significantly less likely to agree with intervention in advance care planning by medical/care staff than those working elsewhere. This could be due to their decreased knowledge regarding advance care planning compared to those working elsewhere, as found in the results of this study.

### Advance care planning practices

The practice of advance care planning was associated with physicians’ gender, specialties, and workplace. Although male physicians were less likely than female physicians to support advance care planning or agree it should be provided by medical/care staff, they were more likely to provide advance care planning. Additionally, this result is inconsistent with a previous study [5], and further research is needed to examine the association between physicians’ gender and advance care planning practice.

Physicians who specialized in internal medicine, general medicine, palliative medicine, and surgery were more likely to practice advance care planning. A previous study similarly reported that attitudes and responsibility towards advance care planning differ among physicians according to their specialty [12]. This could be due to differences in the main target diseases and their natural course.

The finding that physicians working in clinics were significantly less likely to practice advance care planning than those working elsewhere may be due to fewer trigger events in clinic settings. The triggers of advance care planning include a change in the patient’s medical condition, the occurrence of serious events, and the need to make choices regarding the treatment or location for end-of-life care [13]. Therefore, physicians working at hospitals might have a better chance to perform advance care planning and consequently feel more necessity to discuss end-of-life care with their patients than those working in clinics. The participation in advance care planning among physicians in primary care is low [14]; therefore, it is necessary to promote advance care planning among physicians working in clinics. Previous studies have reported inconsistent results regarding the association between the practice of advance care planning and the workplace. One study reported that physicians working in hospitals were significantly more likely to have adequate end-of-life discussions with their dying patients [2]; but another study reported that advance care planning engagement or consideration was not associated with whether the physician worked in a hospital [7]. These studies used the same dataset from a previous Japanese national survey; however, the inconsistent results might be based on their different focus: “end-of-life discussion with dying patients” and “advance care planning.” In the current study, we used a definition of “advance care planning” without the limitation of “dying.” Despite inconsistent results between this study and previous studies, the results of the two sensitivity analyses were consistent with the main analysis.

### What this study adds

This study reveals the need to promote advance care planning targeting physicians working in clinics. Primary care physicians in communities are expected to play a central role in the advance care planning process due to their detailed knowledge of their patients’ long-term health conditions, family situations, and local medical and care systems [1, 15]. However, this study revealed contrary results.

To promote advance care planning in clinics, education programs for physicians as well as providing materials to support patients’ education and advance care planning discussions might be helpful [2, 14, 16]. Additionally, it might be necessary to identify and focus on patients with a high risk of deteriorating and dying due to limited time as it inhibits physicians’ practice of advance care planning [5, 16–18].

### Limitations of the study

This study has some limitations. First, although the participants were from randomly selected facilities, the effective response rate was low (28.0%). Self-selection bias may limit the generalizability of these results, as those who responded may be more positive toward advance care planning or more available than non-responders. Second, the respondents may have excluded situations where they may have had end-of-life discussions with family members rather than the patients. In the questionnaire, the definition of advance care planning was described as “discussions about end-of-life medical care with the patients” with an annotation of “If you could not confirm the intention of the patient themselves, do you think the sufficient discussions based on their intention are being held with families or others?” Despite this proviso, some respondents answered that they did not have sufficient discussions because “most patients are unable to discuss their care” or “I mainly discuss with families.” Third, the quality and contents of advance care planning were unclear in this study. Although the definition of advance care planning was specified in the questionnaire, there were no questions regarding the details or elements of advance care planning. Sufficient discussions may have only included limited elements, such as do-not-resuscitate requests. Fourth, there may have been inaccuracies when categorizing physicians’ specialties. Respondents could select multiple specialties, and some physicians selected internal or general medicine as well as surgery, pediatrics, dermatology, or orthopedics. However, these physicians might not have specialized primarily in internal or general medicine, and they may not have treated patients continuously as their primary care physicians. Furthermore, it may not be reasonable to expect physicians categorized as “internal or general physician working in clinics” to discuss end-of-life care with their patients.

## Conclusions

This study found significant associations between physicians’ knowledge, attitudes, and practice of advance care planning and their gender, specialties, and workplace. Physicians working in clinics were less knowledgeable and less likely to practice advance care planning; therefore, targeted interventions are needed to promote physician advance care planning in clinics.

### Supplementary Information

**Additional file 1.**

**Additional file 2.**

**Additional file 3.**

## Data Availability

The data supporting the findings of this study are available from the Ministry of Health, Labour and Welfare but restrictions apply to the availability of these data, which were used under license for the current study, and so are not publicly available. Data are however available from the authors upon reasonable request and with permission of the Ministry of Health, Labour and Welfare.

## Abbreviations

CI: Confidence interval
OR: Odds ratio
