# Supplementary material for: Association between physicians’ characteristics and their knowledge, attitudes, and practices regarding advance care planning: a cross-sectional study
Source: BMC Palliat Care. 2023 Sep 11;22:134. doi: 10.1186/s12904-023-01253-x (PMC10494406; doi:10.1186/s12904-023-01253-x)
Supplement: Supplementary file 1 — Additional file 1. [file 12904_2023_1253_MOESM1_ESM.docx]

**Appendix 1: Details of the original questionnaire**

***1) Knowledge of advance care planning***

Question: “Do you know about advance care planning?”

Answers (select one of followings): (1) Know well, (2) Ever heard but not know well, (3) Do not know.

We re-categorized the responses into “Know well” and “Not know well,” which includes “Ever heard but not know well” and “Do not know.”

***2)*** ***Promotion of advance care planning***

Question: “Do you agree with promoting advance care planning?”

Answers (select one of following): (1) Agree, (2) Disagree, (3) Not sure.

***3) Provision of advance care planning by medical/care staff*.**

Question**:** “Do you think that interventions for promoting advance care planning should be provided by medical and care staffs?”

Answers (select one of following): (1) Necessary, (2) Not necessary, (3) Not sure.

**4) *Advance care planning practices***

Question: “Do you think that sufficient discussions about end-of-life medical care are being held with patients themselves?” (If you are unable to confirm the intention of the patient, do you think that sufficient discussions based on their preferences are being held with families or others?)

Answers (select one of following): (1) Sufficiently, (2) Some extent, (3) Infrequent, (4) I am not involved with patients in end-of-life stage.

We re-categorized the responses into “Practice,” which includes “Sufficiently” and “Some extent,” and “No,” which includes “Infrequent” and “I am not involved with patients in end-of-life stage.”
