# Supplementary material for: Association between physicians’ characteristics and their knowledge, attitudes, and practices regarding advance care planning: a cross-sectional study
Source: BMC Palliat Care. 2023 Sep 11;22:134. doi: 10.1186/s12904-023-01253-x (PMC10494406; doi:10.1186/s12904-023-01253-x)
Supplement: Supplementary file 2 — Additional file 2. [file 12904_2023_1253_MOESM2_ESM.docx]

**Appendix 2: Sensitivity Analysis**

(i) Excluding responders who answered, ‘I am not involved in patients in end-of-life stage.’(n= 1073)

1) Good knowledge of advance care planning

|  | |  | Bivariate analysis | | | | | |  | Multivariable  logistic regression analysis | | | |
| --- | --- | --- | --- | --- | --- | --- | --- | --- | --- | --- | --- | --- | --- |
|  | |  | Yes  (N= 560) | |  | No  (N= 513) | |  |  | OR [95%CI] | | | |
|  | |  | n(%) | |  | n(%) | | p |  |  |  |  |  |
| Sex | | |  | |  |  |  | |  |  |  |  |  |
|  | Male | | 473 | (51.5) |  | 445 | (48.5) | 0.29 |  | 0.92 | [0.61 | - | 1.37] |
| Years of practice | | |  |  |  |  |  | 0.19 |  |  |  |  |  |
|  | 1–15 | | 76 | (55.9) |  | 60 | (44.1) |  |  | 1.00 | (ref) | | |
|  | 16–30 | | 219 | (54.6) |  | 182 | (45.4) |  |  | 1.30 | [0.83 | - | 2.05] |
|  | ≥31 | | 265 | (49.4) |  | 271 | (50.6) |  |  | 1.25 | [0.80 | - | 1.96] |
| Specialty | | |  |  |  |  |  |  |  |  |  |  |  |
|  | Internal medicine | | 285 | (54.2) |  | 241 | (45.8) | 0.20 |  | 1.70 | [1.17 | - | 2.47] |
|  | General medicine | | 61 | (88.4) |  | 8 | (11.6) | <0.001 |  | 7.59 | [3.47 | - | 16.59] |
|  | Palliative care | | 121 | (93.1) |  | 9 | (6.9) | <0.001 |  | 13.91 | [6.77 | - | 28.60] |
|  | Surgery | | 125 | (59.5) |  | 85 | (40.5) | 0.018 |  | 1.42 | [0.93 | - | 2.16] |
|  | Others | | 174 | (40.9) |  | 251 | (59.1) | <0.001 |  | 0.79 | [0.55 | - | 1.14] |
| Workplace | | |  |  |  |  |  |  |  |  |  |  |  |
|  | Hospital | | 425 | (61.5) |  | 266 | (38.5) | <0.001 |  | 1.00 | (ref) | | |
|  | Clinic | | 105 | (32.2) |  | 221 | (67.8) | <0.001 |  | 0.42 | [0.31 | - | 0.57] |
|  | Nursing home | | 5 | (29.4) |  | 12 | (70.6) | 0.058 |  | 0.32 | [0.11 | - | 0.95] |
|  | Others | | 25 | (64.1) |  | 14 | (35.9) | 0.13 |  | 1.64 | [0.80 | - | 3.35] |

2) Agree that advance care planning should be promoted

|  | |  | Bivariate analysis | | | | | | |  | Multivariable  logistic regression analysis | | | |
| --- | --- | --- | --- | --- | --- | --- | --- | --- | --- | --- | --- | --- | --- | --- |
|  | |  | Yes  (N= 855) | |  | No/Not sure  (N= 218) | |  | |  | OR [95%CI] | | | |
|  | |  | n (%) | |  | n (%) | | p | |  |  |  |  |  |
| Sex | | |  | |  |  |  | | 0.007 |  |  |  |  |  |
|  | Male | | 719 | (78.3) |  | 199 | (21.7) |  |  |  | 0.49 | [0.29 | - | 0.84] |
| Years of practice | | |  |  |  |  |  |  | 0.020 |  |  |  |  |  |
|  | 1–15 | | 120 | (88.2) |  | 16 | (11.8) |  |  |  | 1.00 | (ref) | | |
|  | 16–30 | | 320 | (79.8) |  | 81 | (20.2) |  |  |  | 0.60 | [0.33 | - | 1.08] |
|  | ≥31 | | 415 | (77.4) |  | 121 | (22.6) |  |  |  | 0.62 | [0.34 | - | 1.12] |
| Specialty | | |  |  |  |  |  |  |  |  |  |  |  |  |
|  | Internal medicine | | 433 | (82.3) |  | 93 | (17.7) |  | 0.035 |  | 1.72 | [1.12 | - | 2.66] |
|  | General medicine | | 62 | (89.9) |  | 7 | (10.1) |  | 0.030 |  | 1.77 | [0.78 | - | 4.02] |
|  | Palliative care | | 116 | (89.2) |  | 14 | (10.8) |  | 0.004 |  | 1.69 | [0.91 | - | 3.16] |
|  | Surgery | | 173 | (82.4) |  | 37 | (17.6) |  | 0.28 |  | 1.37 | [0.84 | - | 2.23] |
|  | Others | | 317 | (74.6) |  | 108 | (25.4) |  | <0.001 |  | 0.98 | [0.64 | - | 1.51] |
| Workplace | | |  |  |  |  |  |  |  |  |  |  |  |  |
|  | Hospital | | 590 | (85.4) |  | 101 | (14.6) |  | <0.001 |  | 1.00 | (ref) | | |
|  | Clinic | | 216 | (66.3) |  | 110 | (33.7) |  | <0.001 |  | 0.38 | [0.27 | - | 0.54] |
|  | Nursing home | | 16 | (94.1) |  | 1 | (5.9) |  | 0.22 |  | 2.90 | [0.38 | - | 22.41] |
|  | Others | | 33 | (84.6) |  | 6 | (15.4) |  | 0.44 |  | 0.99 | [0.40 | - | 2.47] |

3) Agree that advance care planning should be provided by medical and care staff

|  | |  | Bivariate analysis | | | | | | |  | Multivariable  logistic regression analysis | | | |
| --- | --- | --- | --- | --- | --- | --- | --- | --- | --- | --- | --- | --- | --- | --- |
|  | |  | Yes  (N= 909) | |  | No/Not sure  (N= 164) | |  | |  | OR [95%CI] | | | |
|  | |  | n (%) | |  | n (%) | | p | |  |  |  |  |  |
| Sex | | |  | |  |  |  | | 0.002 |  |  |  |  |  |
|  | Male | | 765 | (83.3) |  | 153 | (16.7) |  |  |  | 0.38 | [0.20 | - | 0.74] |
| Years of practice | | |  |  |  |  |  |  | 0.21 |  |  |  |  |  |
|  | 1–15 | | 122 | (89.7) |  | 14 | (10.3) |  |  |  | 1.00 | (ref) | | |
|  | 16–30 | | 339 | (84.5) |  | 62 | (15.5) |  |  |  | 0.69 | [0.37 | - | 1.31] |
|  | ≥31 | | 448 | (83.6) |  | 88 | (16.4) |  |  |  | 0.77 | [0.41 | - | 1.43] |
| Specialty | | |  |  |  |  |  |  |  |  |  |  |  |  |
|  | Internal medicine | | 452 | (85.9) |  | 74 | (14.1) |  | 0.28 |  | 1.28 | [0.80 | - | 2.06] |
|  | General medicine | | 66 | (95.7) |  | 3 | (4.3) |  | 0.009 |  | 3.36 | [1.03 | - | 10.97] |
|  | Palliative care | | 122 | (93.8) |  | 8 | (6.2) |  | 0.002 |  | 2.42 | [1.12 | - | 5.26] |
|  | Surgery | | 185 | (88.1) |  | 25 | (11.9) |  | 0.13 |  | 1.42 | [0.82 | - | 2.47] |
|  | Others | | 340 | (80.0) |  | 85 | (20.0) |  | <0.001 |  | 0.72 | [0.45 | - | 1.15] |
| Workplace | | |  |  |  |  |  |  |  |  |  |  |  |  |
|  | Hospital | | 602 | (87.1) |  | 89 | (12.9) |  | 0.003 |  | 1.00 | (ref) | | |
|  | Clinic | | 253 | (77.6) |  | 73 | (22.4) |  | <0.001 |  | 0.66 | [0.46 | - | 0.96] |
|  | Nursing home | | 16 | (94.1) |  | 1 | (5.9) |  | 0.49 |  | 2.68 | [0.35 | - | 20.80] |
|  | Others | | 38 | (97.4) |  | 1 | (2.6) |  | 0.025 |  | 6.41 | [0.86 | - | 47.78] |

OR: Odds Ratio, CI: Confidence Interval

4) Advance care planning practice

|  | |  | Bivariate analysis | | | | | | |  | Multivariable  logistic regression analysis | | | |
| --- | --- | --- | --- | --- | --- | --- | --- | --- | --- | --- | --- | --- | --- | --- |
|  | |  | Practice  (N= 800) | |  | No  (N= 273) | |  | |  | OR [95%CI] | | | |
|  | |  | n (%) | |  | n (%) | | p | |  |  |  |  |  |
| Sex | | |  | |  |  |  | | 0.477 |  |  |  |  |  |
|  | Male | | 688 | (75.0) |  | 230 | (25.1) |  |  |  | 1.22 | [0.77 | - | 1.93] |
| Years of practice | | |  |  |  |  |  |  | 0.007 |  |  |  |  |  |
|  | 1–15 | | 113 | (83.1) |  | 23 | (16.9) |  |  |  | 1.00 | (ref) | | |
|  | 16–30 | | 307 | (76.6) |  | 94 | (23.4) |  |  |  | 0.80 | [0.46 | - | 1.41] |
|  | ≥31 | | 380 | (70.9) |  | 156 | (29.1) |  |  |  | 0.64 | [0.37 | - | 1.11] |
| Specialty | | |  |  |  |  |  |  |  |  |  |  |  |  |
|  | Internal medicine | | 419 | (79.7) |  | 107 | (20.3) |  | <0.001 |  | 2.07 | [1.34 | - | 3.21] |
|  | General medicine | | 64 | (92.8) |  | 5 | (7.3) |  | <0.001 |  | 3.09 | [1.18 | - | 8.09] |
|  | Palliative care | | 125 | (96.2) |  | 5 | (3.9) |  | <0.001 |  | 6.61 | [2.57 | - | 17.04] |
|  | Surgery | | 180 | (85.7) |  | 30 | (14.3) |  | <0.001 |  | 2.10 | [1.25 | - | 3.52] |
|  | Others | | 258 | (60.7) |  | 167 | (39.3) |  | <0.001 |  | 0.63 | [0.41 | - | 0.97] |
| Workplace | | |  |  |  |  |  |  |  |  |  |  |  |  |
|  | Hospital | | 591 | (85.5) |  | 100 | (14.5) |  | <0.001 |  | 1.00 | (ref) | | |
|  | Clinic | | 167 | (51.2) |  | 159 | (48.8) |  | <0.001 |  | 0.24 | [0.17 | - | 0.34] |
|  | Nursing home | | 14 | (82.4) |  | 3 | (17.6) |  | 0.58 |  | 0.93 | [0.25 | - | 3.40] |
|  | Others | | 28 | (71.8) |  | 11 | (28.2) |  | 0.69 |  | 0.67 | [0.31 | - | 1.46] |

OR: Odds Ratio, CI: Confidence Interval

(ii) Excluding responders who answered ‘I am not involved in patients in end-of-life stage’, and include only responders who were specialized internal medicine or general medicine. (n= 567)

1) Good knowledge of advance care planning

|  | |  | Bivariate analysis | | | | | |  | Multivariable  logistic regression analysis | | | |
| --- | --- | --- | --- | --- | --- | --- | --- | --- | --- | --- | --- | --- | --- |
|  | |  | Yes  (N= 323) | |  | No  (N= 244) | |  |  | OR [95%CI] | | | |
|  | |  | n (%) | |  | n (%) | | p |  |  |  |  |  |
| Sex | | |  | |  |  |  | 0.80 |  |  |  |  |  |
|  | Male | | 283 | (57.2) |  | 212 | (42.8) |  |  | 1.31 | [0.77 | - | 2.24] |
| Years of practice | | |  |  |  |  |  | 0.041 |  |  |  |  |  |
|  | 1–15 | | 53 | (66.3) |  | 27 | (33.8) |  |  | 1.00 | (ref) | | |
|  | 16–30 | | 126 | (60.0) |  | 84 | (40.0) |  |  | 0.80 | [0.46 | - | 1.40] |
|  | ≥31 | | 144 | (52.0) |  | 133 | (48.0) |  |  | 0.63 | [0.36 | - | 1.10] |
| Workplace | | |  |  |  |  |  |  |  |  |  |  |  |
|  | Hospital | | 238 | (65.9) |  | 123 | (34.1) | <0.001 |  | 1.00 | (ref) | | |
|  | Clinic | | 71 | (39.9) |  | 107 | (60.1) | <0.001 |  | 0.36 | [0.25 | - | 0.52] |
|  | Nursing home | | 4 | (33.3) |  | 8 | (66.7) | 0.095 |  | 0.26 | [0.08 | - | 0.90] |
|  | Others | | 10 | (62.5) |  | 6 | (37.5) | 0.65 |  | 0.92 | [0.32 | - | 2.60] |

2) Agree that advance care planning should be promoted

|  | |  | Bivariate analysis | | | | | | |  | Multivariable  logistic regression analysis | | | |
| --- | --- | --- | --- | --- | --- | --- | --- | --- | --- | --- | --- | --- | --- | --- |
|  | |  | Yes  (N= 468) | |  | No/Not sure  (N= 99) | |  | |  | OR [95%CI] | | | |
|  | |  | n (%) | |  | n (%) | | p | |  |  |  |  |  |
| Sex | | |  | |  |  |  | | 0.029 |  |  |  |  |  |
|  | Male | | 402 | (81.2) |  | 93 | (18.8) |  |  |  | 0.47 | [0.19 | - | 1.14] |
| Years of practice | | |  |  |  |  |  |  | 0.12 |  |  |  |  |  |
|  | 1–15 | | 72 | (90.0) |  | 8 | (10.0) |  |  |  | 1.00 | (ref) | | |
|  | 16–30 | | 174 | (82.9) |  | 36 | (17.1) |  |  |  | 0.62 | [0.27 | - | 1.43] |
|  | ≥31 | | 222 | (80.1) |  | 55 | (19.9) |  |  |  | 0.66 | [0.29 | - | 1.50] |
| Workplace | | |  |  |  |  |  |  |  |  |  |  |  |  |
|  | Hospital | | 318 | (88.1) |  | 43 | (11.9) |  | <0.001 |  | 1.00 | (ref) | | |
|  | Clinic | | 126 | (70.8) |  | 52 | (29.2) |  | <0.001 |  | 0.35 | [0.22 | - | 0.55] |
|  | Nursing home | | 11 | (91.7) |  | 1 | (8.3) |  | 0.70 |  | 1.41 | [0.18 | - | 11.31] |
|  | Others | | 13 | (81.3) |  | 3 | (18.8) |  | 0.75 |  | 0.65 | [0.18 | - | 2.41] |

3) Agree that advance care planning should be provided by medical and care staff

|  | |  | Bivariate analysis | | | | | | |  | Multivariable  logistic regression analysis | | | |
| --- | --- | --- | --- | --- | --- | --- | --- | --- | --- | --- | --- | --- | --- | --- |
|  | |  | Yes  (N= 491) | |  | No/Not sure  (N= 76) | |  | |  | OR [95%CI] | | | |
|  | |  | n (%) | |  | n (%) | | p | |  |  |  |  |  |
| Sex | | |  | |  |  |  | | 0.085 |  |  |  |  |  |
|  | Male | | 424 | (85.7) |  | 71 | (14.3) |  |  |  | 0.44 | [0.17 | - | 1.16] |
| Years of practice | | |  |  |  |  |  |  | 0.11 |  |  |  |  |  |
|  | 1–15 | | 74 | (92.5) |  | 6 | (7.5) |  |  |  | 1.00 | (ref) | | |
|  | 16–30 | | 175 | (83.3) |  | 35 | (16.7) |  |  |  | 0.45 | [0.18 | - | 1.13] |
|  | ≥31 | | 242 | (87.4) |  | 35 | (12.6) |  |  |  | 0.72 | [0.29 | - | 1.83] |
| Workplace | | |  |  |  |  |  |  |  |  |  |  |  |  |
|  | Hospital | | 320 | (88.6) |  | 41 | (11.4) |  | 0.058 |  | 1.00 | (ref) | | |
|  | Clinic | | 144 | (80.9) |  | 34 | (19.1) |  | 0.007 |  | 0.54 | [0.33 | - | 0.91] |
|  | Nursing home | | 11 | (91.7) |  | 1 | (8.3) |  | 1.00 |  | 1.35 | [0.17 | - | 10.90] |
|  | Others | | 16 | (100.0) |  | 0 | (0.0) |  | 0.15 |  | 1.00 | [1.00 | - | 1.00] |

OR: Odds Ratio, CI: Confidence Interval

4) Advance care planning practice

|  | |  | Bivariate analysis | | | | | | |  | Multivariable  logistic regression analysis | | | |
| --- | --- | --- | --- | --- | --- | --- | --- | --- | --- | --- | --- | --- | --- | --- |
|  | |  | Perform  (N= 458) | |  | No/Not sure  (N= 109) | |  | |  | OR [95%CI] | | | |
|  | |  | n (%) | |  | n (%) | | p | |  |  |  |  |  |
| Sex | | |  | |  |  |  | |  |  |  |  |  |  |
|  | Male | | 400 | (80.8) |  | 95 | (19.2) |  | 0.96 |  | 1.36 | [0.69 | - | 2.67] |
| Years of practice | | |  |  |  |  |  |  | 0.045 |  |  |  |  |  |
|  | 1–15 | | 72 | (90.0) |  | 8 | (10.0) |  |  |  | 1.00 | (ref) | | |
|  | 16–30 | | 171 | (81.4) |  | 39 | (18.6) |  |  |  | 0.52 | [0.22 | - | 1.18] |
|  | ≥31 | | 215 | (77.6) |  | 62 | (22.4) |  |  |  | 0.48 | [0.21 | - | 1.09] |
| Workplace | | |  |  |  |  |  |  |  |  |  |  |  |  |
|  | Hospital | | 318 | (88.1) |  | 43 | (11.9) |  | <0.001 |  | 1.00 | (ref) | | |
|  | Clinic | | 119 | (66.9) |  | 59 | (33.1) |  | <0.001 |  | 0.28 | [0.18 | - | 0.45] |
|  | Nursing home | | 9 | (75.0) |  | 3 | (25.0) |  | 0.61 |  | 0.42 | [0.11 | - | 1.62] |
|  | Others | | 12 | (75.0) |  | 4 | (25.0) |  | 0.55 |  | 0.42 | [0.13 | - | 1.38] |

OR: Odds Ratio, CI: Confidence Interval, ref: reference
